# The Moderating Role of Parenting Dimensions in the Association between Traditional or Cyberbullying Victimization and Mental Health among Adolescents of Different Sexual Orientation

**DOI:** 10.3390/ijerph18062867

**Published:** 2021-03-11

**Authors:** Ann DeSmet, Maddalena Rodelli, Michel Walrave, Gwendolyn Portzky, Eva Dumon, Bart Soenens

**Affiliations:** 1 Faculty of Psychology and Educational Sciences, Université Libre de Bruxelles, 1050 Brussels, Belgium; 2 Department of Communication Studies, Faculty of Social Sciences, Antwerp University, 2000 Antwerp, Belgium; Michel.Walrave@uantwerpen.be; 3 Department of Philosophy, Pedagogy and Applied Psychology, Faculty of Psychology, Sociology, University of Padua, 35122 Padua, Italy; Maddalena.Rodelli@gmail.com; 4 Flemish Centre of Expertise in Suicide Prevention (VLESP), Faculty of Medicine and Health Sciences, Ghent University, 9000 Ghent, Belgium; Gwendolyn.Portzky@ugent.be (G.P.); Eva.Dumon@ugent.be (E.D.); 5 Department of Developmental, Personality and Social Psychology, Faculty of Psychology and Educational Sciences, Ghent University, 9000 Ghent, Belgium; Bart.Soenens@ugent.be

**Keywords:** sexual orientation, cyberbullying, mental health, suicide, parenting, autonomy support, psychological control

## Abstract

Cyberbullying victimization is associated with mental health problems and reported to occur more in nonheterosexual orientation youth (lesbian, gay, bisexual, and questioning (LGBQ)) than among heterosexual youth. Parental support may protect against mental health problems after being victimized, but nonsupportive parental influences may also exacerbate harm. This study investigated whether parenting dimensions (autonomy support, psychological control) moderated the associations between bullying victimization and mental health problems among heterosexual and LGBQ adolescents. An anonymous survey was completed by 1037 adolescents (M age = 15.2 ± 1.9, 50% female). Regression analyses examined associations between victimization, sexual orientation, and mental health problems, and investigated the moderating role of parenting. Both forms of victimization were associated with higher mental health problems. LGBQ youth experienced more depressive symptoms and suicidal ideation than heterosexual youth. Lower levels of parental psychological control and higher levels of autonomy support were related to having fewer mental health problems. However, perceived autonomy support appeared less protective when adolescents experienced more frequent victimization. Moreover, parental psychological control was related to heightened risk for suicidal plans specifically among LGBQ youth and also exacerbated the association between cyberbullying victimization and stress among LGBQ youth. These findings underscore the need to address parenting in whole-school antibullying and mental health promotion programs.

## 1. Introduction

Bullying is commonly defined as an intentional act to hurt, socially isolate, or cause distress to a victim who has less power than the perpetrator, and that results in repeated harm [1]. Cyberbullying is a form of bullying that takes place via digital media, such as social networking sites or text messaging. Cyberbullying victimization (15%) is less prevalent than victimization through traditional bullying, which does not use digital media (35%). It can however result in severe distress [2,3,4] and appears even more strongly associated with suicidal ideation than traditional bullying victimization [5]. Victims of either traditional bullying or cyberbullying can experience a multitude of mental health problems, such as higher levels of depressive symptoms, stress, anxiety, loneliness, substance abuse, somatic symptoms, and suicidal ideation and lower levels of self-esteem, academic achievement, and life satisfaction [6,7].

Youth of lesbian, gay, bisexual, and questioning (LGBQ) sexual orientation are at increased risk of experiencing victimization through both types of bullying [8,9,10,11,12,13,14,15,16]. To lower the risk of poor mental health when adolescents are faced with bullying, protective factors need to be identified and strengthened. Parents are likely to play an important protective role here because the quality of their parenting style can affect adolescents’ resilience or susceptibility to harmful contextual influences [17]. Several studies indeed have demonstrated the protective role of parental and family support and connectedness against mental health problems associated with bullying victimization [18], in particular among LGBQ youth [19,20,21,22].

LGBQ youth may suffer from issues with disclosure and self-acceptance of their sexual orientation. Parental support can make it easier for LGBQ youth to turn to their parents with their concerns and feelings, and receive more personally relevant parental advice [23]. For example, in [24], lower family support moderated the relationship between homophobic bullying and emotional distress. A limitation of existing research is that parenting quality has been operationalized with broad indices of various parenting practices and styles. Investigating specific parenting dimensions rather than family support in general can provide more concrete levers for interventions to improve parenting in relation to youth mental health [25].

This study addresses the role of autonomy-supportive and psychologically controlling parenting dimensions, two constructs that have increasingly received attention for their role in adolescents’ psychosocial adjustment [26,27,28]. Autonomy-supportive parents allow and help their children to make decisions that are in line with their children’s own values and interests [29]. They provide choices, encourage initiative, and display a genuine interest in their children’s feelings and thoughts. In contrast, psychological control is characteristic of parents who try to force their children to act in accordance with the parents’ standards and needs, thereby largely ignoring the children’s own needs and values [30]. These parents use pressuring and manipulative strategies (e.g., ignoring children by giving them the silent treatment, inducing guilt and shame, and making approval conditional of their behavior).

The effects of autonomy-supportive and psychologically controlling parenting on adolescents’ psychosocial adjustment have been found to be largely opposite to each other [31,32,33,34]. In [35,36,37], autonomy-supportive parenting mainly predicted positive adjustment outcomes and was positively related to children’s and adolescents’ well-being and socio-emotional competence. Psychologically controlling parenting has been found to primarily predict ill-being and maladjustment [38], including depressive symptoms and anxiety [25,31,39,40]. Both parenting constructs, however, have a distinct, unique value in predicting specific adolescent outcomes, and their effects should be studied separately [41].

Although some research has examined the role of parenting in predicting bullying involvement [42], to our knowledge, no study has yet examined the role of parenting in protecting adolescents from mental health harm that may be experienced from bullying victimization. This is important as mental health problems related to bullying can have a negative impact on well-being that lasts into adulthood [43]. We expect that autonomy-supportive parenting will make adolescents feel more understood and, as such, will be associated with less mental health problems, whereas parental psychological control may involve parental dismissal of adolescents’ concerns and negative emotions [40]. As such, adolescents experiencing more parental psychological control might suffer more from exposure to victimization.

Moreover, these protective and exacerbating associations could be more pronounced among LGBQ than among non-LGBQ youth. To our knowledge, no study has investigated differences in the protective role of parenting for LGBQ and non-LGBQ youth. In [44], autonomy-supportive parenting was found to increase disclosure and self-acceptance of sexual orientation, indicating that LGBQ youth feel better understood within an autonomy-supportive parenting climate. Accordingly, LGBQ youth may benefit even more (compared with heterosexual youth) from parental autonomy support in terms of alleviating the effects of victimization on mental health problems. In contrast, autonomy-suppressing (controlling) parenting has been found to predict suppression of individuals’ sexual orientation [45], indicating that controlling parenting contributes to a climate in which nontraditional sexual orientations are not accepted. Because of this lack of acceptance, the exacerbating effects of psychological control on the victimization–mental distress association may be even more pronounced among LGBQ youth than among their heterosexual peers.

This study aimed to investigate the protective or exacerbating roles of autonomy-supportive and psychologically controlling parenting in mental health problems after being victimized by traditional bullying or cyberbullying. We furthermore examined these roles among LGBQ and non-LGBQ youth. The following research questions were addressed as main effects: (1) What is the association between traditional bullying and cyberbullying victimization with mental health problems (depressive symptoms, anxiety, stress, suicidal ideation, suicide plans, and attempts)? (2) Do LGBQ youth experience more mental health problems than non-LGBQ youth? (3) What is the association of autonomy-relevant parenting with these mental health problems? The following interaction effects were examined: (4) What is the protective or exacerbating association of autonomy-supportive and psychologically controlling parenting with mental health outcomes when faced with traditional or cyberbullying victimization (two-way)? (5) Do these (protective or exacerbating) associations of parenting with mental health outcomes differ between LGBQ and non-LGBQ youth (three-way)?

We hypothesized that (1) both traditional and cyberbullying victimization would be associated with more mental health problems (H1); (2) LGBQ youth would experience more mental health problems than non-LGBQ youth (H2); (3) parental autonomy support would be associated with less negative mental health outcomes, and psychologically controlling parenting would be associated with more mental health problems (H3); (4) autonomy-supportive parenting would buffer associations between traditional bullying or cyberbullying victimization and mental health problems, while psychologically controlling parenting would exacerbate these associations (H4); (5) and these protective and exacerbating associations would be more pronounced among LGBQ than among non-LGBQ youth (H5). This study took place in Belgium, where equal rights of individuals regardless of their sexual orientation are strongly advocated. Consequently, Belgium scores high on the Rainbow Index (an annual benchmarking tool that ranks countries on their LGBTI (lesbian, gay, bisexual, transgender and intersex) equality laws and policies) for its support for equal rights for members of the LGBQ community. Despite legislative and administrative equal rights, the LGBQ community’s equal opportunities are still sometimes hindered by stigmatization and victimization, and subsequent mental health problems. Schools are an important setting to reduce stigmatization from an early age, to empower members of the LGBQ community, and for all citizens to adopt an awareness and nondiscriminatory attitude towards people with a nonheterosexual orientation.

## 2. Materials and Methods

### 2.1. Participants and Design

A random sample of secondary schools was selected (*n* = 26) in Flanders, Belgium (a region with 6 million inhabitants), based on a government database of schools. Schools that provided technical/arts, vocational, or academic track education were included. Schools that only provided special needs education were excluded. Schools were first contacted through an e-mail containing information about the study and asking for their participation, followed up by a phone call to ensure the information was well received and understood and to confirm or decline their participation and make practical arrangements. Eight schools (response rate, 31%) participated in which data were collected from grades 7 to 12 (aged 12–18). The other 18 schools declined to participate, the main reason for not participating being lack of time. Education is mainly regulated in Belgium by two umbrella networks: community education, which traditionally includes state-run schools, and “free” education, which comprises most often Catholic schools. Schools from both networks are subsidized by the government and adhere to the same learning objectives, which they can each translate into specific learning plans according to their (ideological) preferences. Three participating schools were community schools, and five were part of the “free” network. Four participating schools provided all forms of educational tracks (academic, technical/arts, vocational), one did not provide the academic track, and three did not provide technical/vocational education.

Data collection took place at school, during 1 class hour, using an anonymous paper-and-pencil survey. Students were told they were under no obligation to participate and could withdraw at any time. Adolescents provided active informed consent, and parents provided passive informed consent. Five students declined to participate (99.5% response rate), and none of the parents declined to provide consent (100% response rate). Students were assured that their responses would be confidential and that no information would be shared with teachers, parents, or fellow students. The study received approval from the Ethics Committee of the Ghent University Hospital.

### 2.2. Measurements

#### 2.2.1. Sociodemographic Information

Sociodemographic variables included age, gender, type of education, country of birth, family situation (all derived from the Health Behavior in School-Aged Children (HBSC) study [46]), and family affluence, measured by the validated self-report scale for adolescents “Family Affluence Scale” (FAS) [47].

#### 2.2.2. Sexual Orientation

Sexual orientation was measured using the question “Who do you usually fall in love with?” Adolescents could answer with “girls,” “boys,” “both girls and boys,” or “I am not sure.” In combination with adolescents’ gender, sexual orientation was determined as heterosexual (non-LGBQ) or lesbian, gay, bisexual, or questioning (LGBQ). The study did not focus on gender identity and hence did not identify transgender youth as a specific group.

#### 2.2.3. Bullying Involvement

Adolescents received a definition of bullying, setting it apart from non-intentional acts and fights between youth of equal power [48], prior to receiving questions on their bullying involvement. Bullying was defined as follows: “We call something bullying when people say or do mean things, when they have the intention to make others feel bad, and when the person who is bullied has a difficult time defending himself or herself. We do not call it bullying when it is about teasing each other or having a row.” Traditional bullying victimization was measured by a single item asking about victimization experience in the past 6 months and rated on a 5-point rating scale (range 0–4) [48]. Cyberbullying victimization was measured by a single item asking about their victimization “via text message or Internet” in the past 6 months and rated on a 5-point rating scale. Given the skewed distribution, both victimization scales were dichotomized into “at least once” and “never” in the past 6 months when used as a dependent variable. Participants indicated perpetration experiences in the same format as for victimization. Perpetration rates are reported in the sample information but are not the focus of this study.

#### 2.2.4. Mental Health

Mental health problems were measured with the Depression, Anxiety, Stress scale (DASS-21), a validated scale for measuring adolescent mental health outcomes. It consists of 7 items per subscale [49]. Total scores per subscale were used as dependent variables, with each subscale showing high reliability (α depression = 0.90, α anxiety = 0.84, α stress = 0.87).

Suicidal thoughts, plans, and behaviors were measured by three questions: (1) suicidal ideation in the past 6 months (having had suicidal thoughts, 5-point Likert scale), (2) suicidal plans in the past 6 months (yes/no), and (3) suicide attempts in the past 6 months (yes/no). These questions were adapted from the Flemish HBSC study [46]. Content validity was established via expert consultation with the Expertise Centre for Suicide Prevention and the suicide hotline in Flanders, Belgium. Given the skewed distribution for suicidal ideation, this variable was dichotomized into “never” and “at least once” in the past 6 months.

#### 2.2.5. Autonomy-Supportive and Psychologically Controlling Parenting

The validated Dutch version [50,51,52] of the Autonomy Support Scale from the Perceptions of Parents Scale [53] was used to measure perceived maternal and paternal autonomy support (rated separately). The scale consists of 7 items rated on a 5-point Likert scale (range 1–5, α maternal autonomy support = 0.70, α paternal autonomy support = 0.78, e.g., “My mother/father allows me to decide things for myself”). The validated Dutch version [54,55] of the Psychological Control Scale–Youth Self-Report (PCS-YSR) [30] was used to measure perceived maternal and paternal psychological control, consisting of 8 items (range 1–5, α maternal psychological control = 0.84, α paternal psychological control = 0.87, e.g., “My mother/father is less friendly to me when I don’t see things her/his way”). Given the moderately high correlations between maternal and paternal psychological control (r = 0.54) and between maternal and paternal autonomy support (r = 0.48), maternal and paternal scores were averaged, and these average scores aggregated across both parents were used in the regression analyses.

### 2.3. Analyses

Data were first inspected for a normal distribution based on a visual inspection of the histogram and Q–Q plots. Because suicidal risk did not follow a normal distribution, logistic regression analyses were used to assess direct and moderated associations between sexual orientation, bullying and cyberbullying involvement, parenting, and suicidal risk (suicidal ideation, suicidal plans, suicidal attempts). Linear regression analyses were used to assess direct and moderated associations between sexual orientation, bullying and cyberbullying victimization, parenting and depressive symptoms, anxiety, and stress. Hierarchical (blockwise) entry of predictors was used by first adding sociodemographic, main-effects, and next the interaction terms into the analysis. Models were rerun, removing nonsignificant predictors, to come to a parsimonious model. If several predictors are strongly correlated, the estimates become less trustworthy. Multicollinearity was therefore assessed in collinearity diagnostics checking the variance inflation factor (≤10) and tolerance (≥0.1). Maternal and paternal parenting dimensions were already combined into an aggregated score to reduce potential problems of multicollinearity. No other variables showed multicollinearity. Continuous independent variables were centered on the mean, and the interaction models always also included the main effects of these variables. Interaction variables were created by multiplying the centered scores for the main predictors. To avoid biased results in logistic regressions, cross tabulations were checked for empty combinations of cells or low expected frequencies [56]. The sample size in the study was large compared with the number of predictors, reducing the risk of overfitting the model.

All initial models were corrected for age and gender. Tables in the manuscript show parsimonious results, and the supporting information file shows nonparsimonious model results, including nonsignificant effects (Tables S1–S3 in Supporting Information). All analyses were conducted using SPSS 25. (IBM, Armonk, New York, USA) Graphical representations of moderator analyses were made using PROCESS 2.16.3 for SPSS, and simple slopes analyses for significant moderators were based on parsimonious model results. Graphs for a three-way interaction with one categorical variable (i.e., sexual orientation) were constructed as two-way interaction effects, separately for each level of the categorical variable.

## 3. Results

### 3.1. Descriptive Statistics

The analyzed sample consisted of 1037 adolescents, after removing 25 participants whose answers were incomplete or showed satisficing patterns. The sample consisted of 49.8% females and had a mean age of 15.17 years. The majority of the sample (82.4%) were of high family affluence, which is consistent with the high affluence rate in this region (i.e., 72.7%; HBSC study). The majority (94.0%) were born in Belgium. About two-thirds (64.1%) of the participants lived with their mother and father, whereas one-third lived in different family situations (e.g., coparenthood, single-parent family, raised by grandparents, living in an institution). Less than 10% of the sample reported a different sexual orientation from heterosexual (0.8% lesbian, 1.2% gay, 1.6% bisexual, 3.5% questioning). About 15%−16% reported having been involved in traditional offline bullying (respectively victim, perpetrator), whereas 7%–9% reported having been involved in cyberbullying (resp. victim, perpetrator). The most frequently reported types were “hurtful things about me were sent privately to others” (14.1%); “being socially excluded” (11.4%); “hurtful things about me were sent publicly to others” (8.9%); “hurtful things were sent to me privately via text” (8.7%); “hurtful things were sent to me publicly via social media or websites” (7.7%); “hurtful things were sent to me privately via email/messenger” (7.3%); “unpleasant action, such as virus or breaking into profile” (6.3%); “embarrassing pictures from me were sent to others privately/publicly” (both 4.9%); and “sexual or nude images from me were sent to others, privately or publicly” (resp. 1.6%−1.2%). There were no significant differences between LGBQ youth and heterosexual youth in the cyberbullying types they experienced, with the exception of cyberbullying of sexual nature (sexual/nude pictures), which was experienced more by LGBQ youth than heterosexual youth (F(1, 990) = 0.33, *p* = 0.003 for privately sharing with others; F(1, 989) = 0.23, *p* = 0.008 for publicly sharing with others).

In the past 6 months, 22% of the adolescents had suicidal thoughts at least once, 8.4% had made suicide plans, and 5.8% had attempted suicide. Table 1 shows descriptive information and correlations between variables. Collinearity diagnostics showed that all tolerance values were above 0.1 and Variance Inflation Factor VIF below 10, indicating no multicollinearity among the independent variables. The expected count was >1 for all cells in cross tabulations. Table 2 shows that LGBQ youth perceived significantly lower levels of maternal autonomy support, but not paternal autonomy support (borderline significant), and higher levels of both maternal and paternal psychologically controlling parenting.

Results from these data reported elsewhere showed that LGBQ youth had significantly increased odds compared with non-LGBQ youth to be victimized by traditional bullying, but not by cyberbullying victimization. There were increased odds among LGBQ youth to be a perpetrator of cyberbullying, but not of traditional bullying [57].

### 3.2. Regression Analyses

#### 3.2.1. Main Effects

A first research question investigated whether bullying victimization was associated with mental health outcomes. Both traditional bullying victimization and cyberbullying victimization were positively associated with more mental health problems, confirming Hypothesis 1. As victimization from both types of bullying becomes more frequent, levels of mental health problems also become higher. Associations were significant for all studied mental health outcomes (i.e., depressive symptoms, anxiety, stress, suicidal ideation, plans, and attempts (Table 3 and Table 4)).

LGBQ youth experienced more depressive symptoms and suicidal attempts than non-LGBQ youth (increased odds when corrected for cyberbullying victimization), but they did not show a higher occurrence of other mental health problems than non-LGBQ youth, partially confirming Hypothesis 2 (Table 4).

Perceived parental autonomy support was associated with less mental health problems (i.e., depressive symptoms, anxiety, suicide attempts when correcting for traditional bullying victimization, and with depressive symptoms and anxiety when correcting for cyberbullying victimization), whereas perceived parental psychological control was associated with higher levels of mental health problems in all indicators (Table 3 and Table 4). These findings partially confirmed Hypothesis 3. When youth had a stronger perception that their parents were psychologically controlling, they experienced more mental health problems. When youth more strongly perceived their parents to be autonomy supportive, they experienced fewer depressive symptoms, suicide attempts, anxiety, but showed no differences with other mental health problems in comparison with youth who felt their parents were less autonomy supportive.

#### 3.2.2. Two-Way Interaction Effects between Parenting and Victimization

The moderating role of parenting in mental health problems was next examined for each dimension of parenting and for each type of bullying victimization.

**Figure 1:**
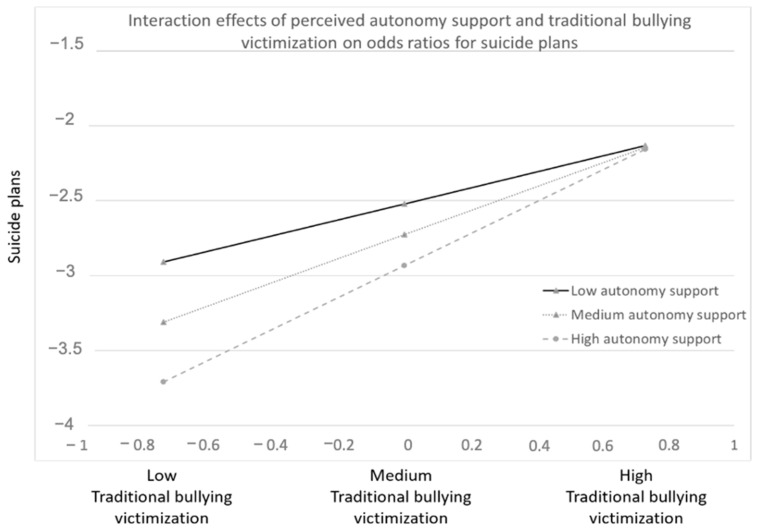
Interactions between traditional bullying victimization, perceived autonomy support, and suicide plans.

**Figure A1:**
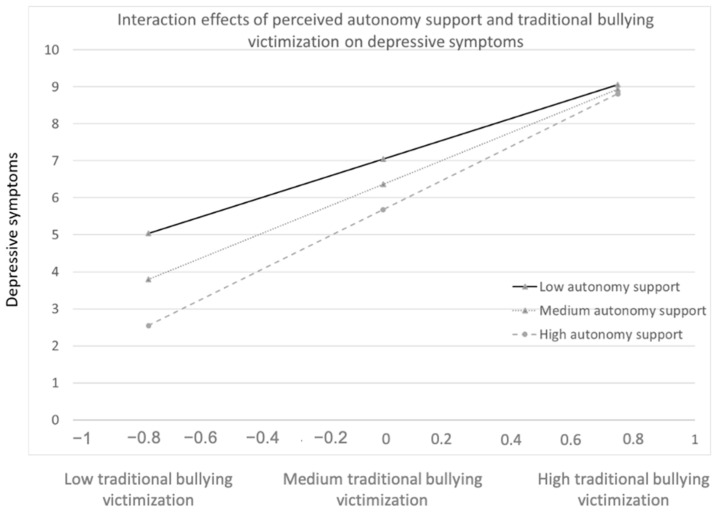
Interactions between autonomy support, traditional bullying victimization, and depressive symptoms.

Two out of six interaction effects between autonomy-supportive parenting and traditional bullying victimization experiences on mental health outcomes were significant. First, at less frequent traditional bullying victimization, lower autonomy support was associated with more suicidal plans than was the case for higher levels of parental autonomy support (Figure 1). As the frequency of traditional bullying victimization increased, this protective association of autonomy support, however, disappeared. Second, a diminishing protective association was also noted for autonomy support with depressive symptoms when faced with traditional bullying victimization (Figure A1 in Appendix A).

**Figure A2:**
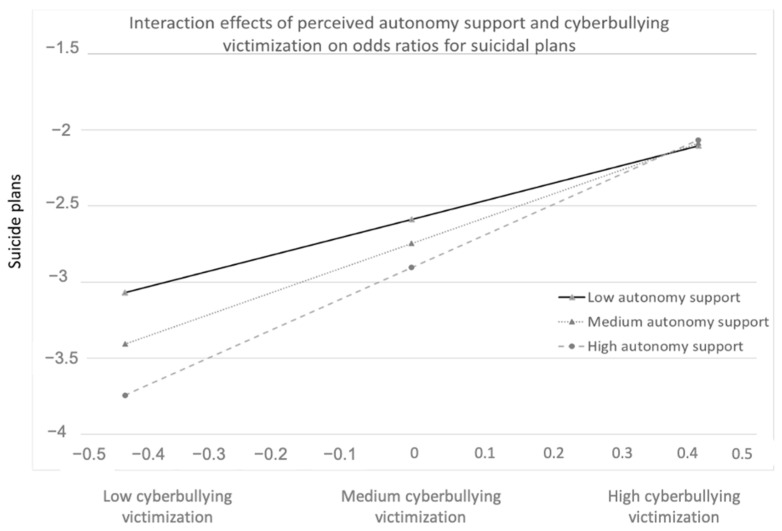
Interactions between autonomy support, cyberbullying victimization, and suicide plans.

**Figure A3:**
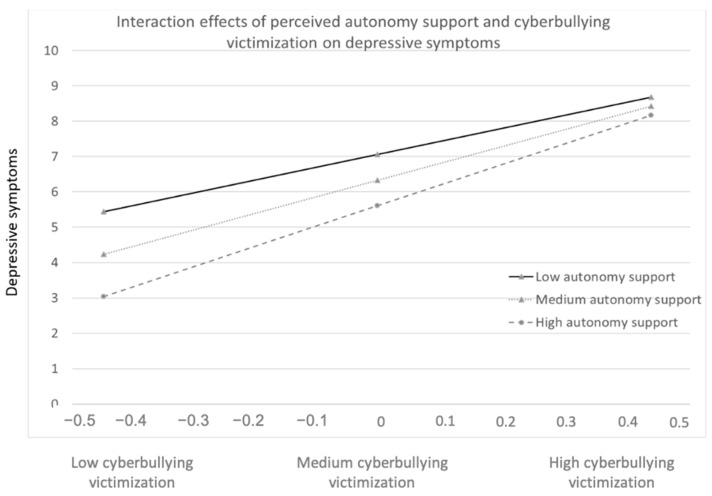
Interactions between autonomy support, cyberbullying victimization, and depressive symptoms.

**Figure A4:**
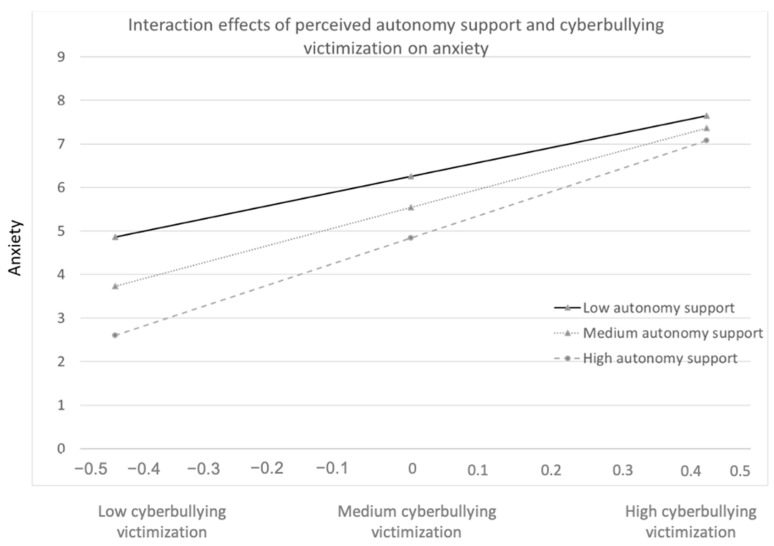
Interactions between autonomy support, cyberbullying victimization, and anxiety.

Three out of six tested interaction effects were significant for cyberbullying victimization. Again, autonomy support was negatively related to suicidal plans and depressive symptoms only at low levels of cyberbullying victimization (Figure A2 and Figure A3 in Appendix A). Moreover, a similar significant interaction effect was found for autonomy support in relation to anxiety when faced with cyberbullying victimization (Figure A4 in Appendix A). These findings do not provide full support for Hypothesis 4: higher parental autonomy support was associated with less mental health problems among adolescents, but for some outcomes only at lower levels of victimization. No two-way interaction effects between victimization and psychologically controlling parenting were found for any mental health outcome (0/12 tested two-way interactions). In sum, autonomy support was associated with fewer mental health problems when faced with traditional or cyberbullying victimization, but only at lower levels of bullying victimization. Psychological control had no protective or exacerbating role in mental health problems when faced with either type of bullying.

#### 3.2.3. Three-Way Interaction Effects between Parenting Dimensions, Victimization, and Sexual Orientation

**Figure 2:**
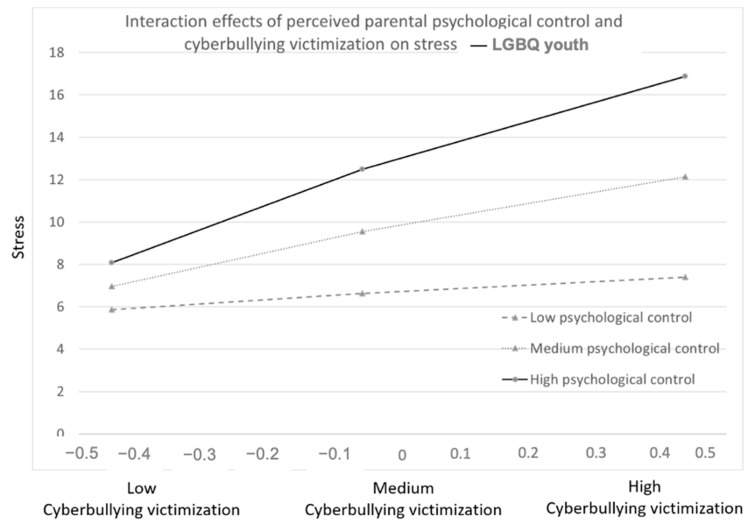
Interaction between cyberbullying victimization, perceived parental psychological control, and stress, among LGBQ youth. Bold: It is to point out that these findings were only found for LGBQ youth.

**Figure A5:**
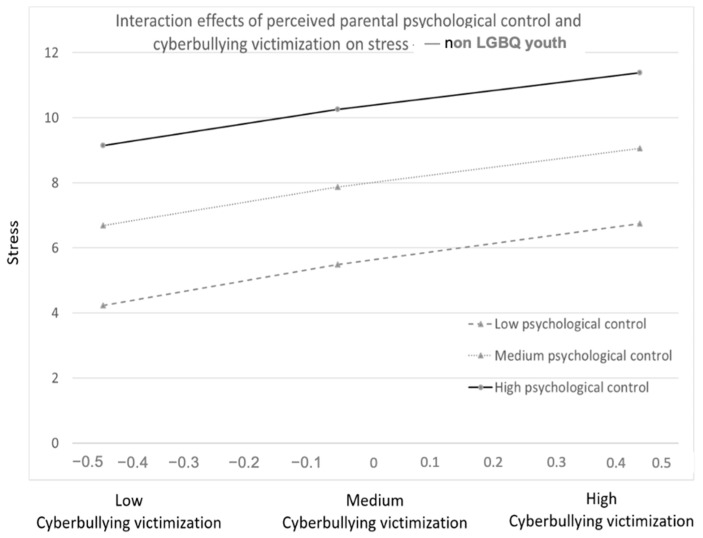
Interactions between psychological control, cyberbullying victimization, and stress among heterosexual (non-LGBQ) youth. Bold: It is to point out that these findings were only found for non-LGBQ youth.

One out of six tested three-way interactions between perceived parental psychological control, sexual orientation, and victimization was significant (Table 4). Among LGBQ youth, perceived psychologically controlling parenting was associated with exacerbated effects of cyberbullying victimization on stress (Figure 2). This interaction effect was not significant among non-LGBQ youth (Figure A5 in Appendix A). This result provided only partial confirmation of Hypothesis 5: parental psychological control was only associated with exacerbated mental health problems among LGBQ youth for cyberbullying victimization in relation to one outcome. Autonomy support did not provide a stronger protection against mental health problems in LGBQ than in non-LGBQ youth.

## 4. Discussion

This study investigated the moderating role of autonomy-supportive and psychologically controlling parenting in the association between youngsters’ bullying experiences, sexual orientation, and mental health problems, including depressive symptoms, anxiety, stress, suicidal thoughts, plans, and behavior.

We first explored the main effects of victimization, sexual orientation, and parenting on mental health outcomes. Youth who experienced more victimization by traditional bullying or cyberbullying also experienced more mental health problems on all measured outcomes, consistent with conclusions from systematic reviews and meta-analyses [6,7]. LGBQ youth also experienced more mental health problems, such as depressive symptoms, anxiety, stress, and suicide attempts, than non-LGBQ youth. Such differences have been highlighted in earlier studies, which showed that LGBQ youth displayed heightened levels of depression [58,59], anxiety, stress, [59], and suicide attempts [60]. Our study also documented the main effects of parental autonomy support and psychological control on adolescent mental health outcomes. Previous studies have shown that psychological control is associated with more depression, anxiety, stress, and suicidal risk [31,32], and that higher perceived parental autonomy support is associated with lower levels of some mental health problems, such as depressive symptoms, anxiety, and suicidal attempts [25,35]. Our findings are consistent with this literature. In general, psychological control has shown stronger associations with mental health problems than autonomy-supportive parenting, which is considered to be more predictive of positive well-being rather than mental health problems [38,41]. Our findings support scarce earlier research showing that autonomy support relates negatively to mental health problems even when taking into account the effects of parental psychological control [25,35]. In particular, the associations with suicidal ideation and behavior are noteworthy. Few studies to date have investigated the role of autonomy-supportive parenting in adolescent suicidal ideation and behavior (see [61] for an exception). Although a low perceived level of autonomy support appears to be a risk factor for suicidal ideation and behavior, the associations between high psychologically controlling parenting and suicidal risk are still stronger than those with autonomy support. Psychological control is assumed to contribute to higher levels of self-criticism [62,63,64,65] and hopelessness [61], which can lead to a greater risk for suicidal ideation and behaviors.

The primary and most innovative goal of this study, however, was to examine the moderating role of autonomy-supportive and psychologically controlling parenting in the risks associated with bullying victimization among youth of heterosexual and nonheterosexual orientation. The dimensions of autonomy-supportive and psychologically controlling parenting moderated some of the associations between victimization or sexual orientation and mental health problems. Parental autonomy support moderated the relationship between traditional bullying and cyberbullying victimization and mental health problems. Perceived parental autonomy support was associated with lower mental health problems, but this association decreased as victimization by traditional bullying and cyberbullying became more frequent. Although, contrary to our hypothesis, this finding is consistent with a study that documented a protective role of family support against poorer mental health and more suicidal thoughts at lower levels of traditional bullying victimization, it is not so at higher levels [22]. At higher levels of bullying victimization, more may be needed to support adolescent mental health than an autonomy-supportive parental style alone. Possibly, in such challenging circumstances, autonomy-supportive parenting needs to be combined with the provision of an adequate structure (e.g., [23]). Apart from recognizing the adolescent’s feelings and leaving room for independent problem-solving, parents may need to also provide help and offer assistance (for instance, suggesting different ways in which the adolescent and/or the parent can deal with the problem) when confronted with serious victimization. As such, an important goal for future research is to examine the interplay between autonomy support and the structure provided by parents in alleviating mental health problems associated with victimization.

Perceived parental psychological control was associated with higher stress among victims of cyberbullying, but only among LGBQ youth: LGBQ youth experienced more stress when cyberbullying victimization was more frequent, and their stress levels increased even more when perceiving higher levels of parental psychological control. This suggests that perceived parental psychological control can exacerbate problems associated with cyberbullying victimization, in particular among LGBQ youth. LGBQ youth may experience additional stress in forming an identity that deviates from the norm defined by the majority. Parental psychological control may then make it even harder to establish a positive sense of self, and has indeed been associated with difficulties among youth to accept their nontraditional sexual orientation, as indicated by more internalized homophobia [66,67] and stronger discrepancies between an individual’s explicit and implicit sexual orientation [45].

Overall, our findings indicate that, while the effects of psychologically controlling parenting were generally detrimental, the effects of autonomy-supportive parenting were somewhat mixed. Although autonomy support did not play the anticipated buffering role against all forms of mental health problems after victimization, this parenting dimension did display systematic main effects and direct protective associations with mental health.

Our findings can provide some recommendations for mental health promotion and suicide prevention practice. Current suicide prevention interventions are most often conducted in school or in-patient settings [68], and often do not involve parents or focus on the individual, whereas interventions that also involve parents have shown to be effective in reducing adolescent suicidal risk [68,69]. Whole-school antibullying programs have paid attention to the protective role of autonomy-supportive and caring parenting [70], but especially in cyberbullying, there is still a proliferation of initiatives that do not actively involve parents [71] or where restrictive parental mediation is used [72]. Parents can, however, have a crucial role in supporting their children’s mental health when faced with peer victimization, by not being psychologically controlling and using more autonomy-supportive parenting, as indicated by our results. Additionally, providing structure may protect adolescents from harm when faced with more severe forms of bullying victimization. This assumption remains to be tested in future research. Parents tend to become less autonomy supportive when they perceive a higher threat, presumably to handle their own fears and to get a grip on their children’s behavior [23,73]. By doing so, they may worsen mental health problems experienced by their children who are peer-victimized and/or struggle with their sexual identity. Addressing parenting in such programs is vital to promote adolescent mental health and reduce suicidal risk. Some evidence-based programs exist to increase these positive parenting practices. “How-to Parenting Programs” that teach emotional and empathic communication, positive interactions, and consistent responding, for example, had positive effects on increasing autonomy-supportive parenting, parental affiliation, and structure [74]. Including these evidence-based components from programs to increase parenting dimensions in interventions for youth mental health promotion, suicide prevention, and antibullying approaches is clearly indicated.

The study has several limitations. Despite a relatively large total sample, the sample of LGBQ youth was small, which did not allow for detailed analyses by specific sexual minority subgroup (LG/B/Q). It would be a valuable addition to replicate these findings in a larger-population-based sample that allows for further drilling down by specific sexual minority group. Our study focused on sexual orientation and did not look into gender identity and transgender youth. Since transgender youth also experience victimization and mental health problems, with parenting that is high in autonomy support and low in psychological control being a potential source of support for transgender youth [75], research on parenting dimensions in this target group would also be valuable. The study was cross-sectional and, therefore, cannot determine whether autonomy support and psychological control decreased or increased psychological symptoms or vice versa. Lastly, the scale that was used did not tap into all dimensions of autonomy-supportive parenting. Using other scales (e.g., domain-specific scales or scales combining autonomy support and structure) may provide additional direction for parenting programs. Further research on autonomy-supportive parenting in high-adversity situations is also needed. Such future research may also ask participants whether they had outed their sexual orientation to their parents, whether the nature of bullying was homophobic, and whether adolescents had discussed these victimization experiences with their parents as potential moderators or mediators in the associations between parenting dimensions and mental health outcomes, which were not included in our study. A strength of this study was that unlike previous research on LGBQ youth that has often relied on convenience samples limiting the representativeness of the sample (see, e.g., [76]), our study used a population-based sample of adolescents recruited via schools. This enabled us to also include adolescents who were unsure about their sexual orientation or had not yet disclosed their sexual orientation. The study included both parental psychological control and autonomy support and controlled for each other’s influence in the analyses. As such, it was able to show how specific parenting dimensions can protect against mental health problems associated with bullying experiences among LGBQ youth and can provide directions for future mental health promotion and bullying prevention programs.

## 5. Conclusions

Our study showed that traditional bullying, cyberbullying victimization, and LGBQ sexual orientation were associated with higher suicidal risk and other mental health problems. Youth who experienced more victimization by traditional bullying or cyberbullying experienced more mental health problems on all measured outcomes. LGBQ youth also experienced more mental health problems, such as depressive symptoms, anxiety, stress, and suicide attempts, than non-LGBQ youth. Further, LGBQ youth experienced less autonomy support and more psychological control from their parents than heterosexual youth. These between-group differences in perceived parenting are important because, whereas parental psychological control was also associated with more depressive symptoms, anxiety, stress, suicidal ideation, plans, and attempts, higher parental autonomy support was associated with lower depressive symptoms, lower anxiety, and (in some cases) less suicide attempts. Although parental autonomy support was primarily expected to increase well-being, our findings suggest that autonomy support may also protect against serious mental health issues, such as suicide attempts. Parental psychological control appeared to worsen the effects of cyberbullying victimization on stress specifically among LGBQ youth. While autonomy-supportive parenting was expected to play a more protective role, perceived parental autonomy support was found to be less protective against mental health problems in higher-risk situations. Possibly, other forms of support and help (mainly structure) may also be needed when adolescents are confronted with serious forms of victimization.

## Figures and Tables

**Table 1:** Descriptive statistics and correlations of the variables in the study (*n* = 1037).

Variables	M	SD	1	2	3	4	5	6	7	8
**1. Age**	15.17	1.86								
**2. Traditional bullying victimization**	0.26	0.75	−0.07 *							
**3. Cyberbullying victimization**	0.11	0.47	−0.05	0.44 **						
**4. Parental psychological control**	2.06	0.76	0.14 **	0.12 **	0.04					
**5. Parental autonomy support**	3.74	0.69	0.08 *	−0.18 **	−0.17 **	−0.46 **				
**6. Depressive symptoms**	6.18	8.51	0.06	0.31 **	0.25 **	0.30 **	−0.22 **			
**7. Anxiety**	5.44	7.11	0.04	0.27 **	0.24 **	0.28 **	−0.22 **	0.76 **		
**8. Stress**	7.93	8.11	0.06 *	0.21 **	0.18 **	0.29 **	−0.20 **	0.76 **	0.75 **	-
**9. Suicidal thoughts**	0.40	0.88	−0.01	0.30 **	0.25 **	0.24 **	−0.18 **	0.65 **	0.52 **	0.46 **

* *p* < 0.05; ** *p* < 0.01; *** *p* < 0.001.

**Table 2:** Differences in parenting between youth of different sexual orientation.

Parenting Dimensions	LGBQ	Heterosexual	Difference
**Paternal autonomy support**	M = 3.51 ± 0.88	M = 3.72 ± 0.81	F(1, 843) = 3.73, *p* = 0.054
**Maternal autonomy support**	M = 3.53 ± 0.86	M = 3.78 ± 0.74	F(1, 947) = 7.07, *p* = 0.008
**Paternal psychological control**	M = 2.29 ± 0.87	M = 2.04 ± 0.88	F(1, 843) = 4.55, *p* = 0.033
**Maternal psychological control**	M = 2.27 ± 0.89	M = 2.05 ± 0.81	F(1, 947) = 4.33, *p* = 0.038

**Table 3:** Moderation analyses of parenting dimensions in the relationship between traditional bullying victimization and sexual orientation in negative mental health outcomes. Underline: It was used to more clearly indicate what the difference is between the two tables where one shows traditional bullying and the other cyberbullying.

	Depression	Anxiety	Stress	Suicidal Ideation	Suicidal Plan	Suicidal Attempt
Model information (parsimonious model)	F(5, 953) = 46.93 ***; R^2^ = 0.19	F(4, 955) = 49.49 ***; R^2^ = 0.17	F(3, 958) = 55.33 ***; R^2^ = 0.15	−2 LL = 875.21; χ^2^(3) = 143.50 ***; R^2^ = 0.21	−2 LL = 468.89; χ^2^(7) = 79.56 ***; R^2^ = 0.18	−2 LL = 320.89; χ^2^(6) = 102.71 ***; R^2^ = 0.29
	**β (B, Unstandardized SE)**			**Adjusted Odds Ratio (95% CI)**		
Gender	−0.17 (−2.97, 0.50) ***	−0.18 (−2.54, 0.42) ***	−0.18 (−2.89, 0.49) ***	0.36 (0.25; 0.51) ***	0.45 (0.27; 0.75) **	0.24 (0.12; 0.47) ***
Sexual orientation	-	-	-	-	0.55 (0.13; 2.32)	3.43 (1.41; 8.38) **
Traditional victimization	0.30 (3.40, 0.39) ***	0.23 (2.18, 0.29) ***	0.17 (1.87, 0.33) ***	2.07 (1.68; 2.55) ***	2.20 (1.70; 2.83) **	2.17 (1.70; 2.76) **
**Moderation Effects of Parenting Dimensions**						
Parental autonomy support	−0.08 (−1.00, 0.42) *	−0.09 (−0.94, 0.35) **	-	-	0.74 (0.49; 1.14)	0.52 (0.31; 0.88) *
Parental psychological control	0.24 (2.74, 0.38) ***	0.23 (2.18, 0.32) ***	0.29 (3.19, 0.33) ***	2.32 (1.86; 2.90) ***	1.76 (1.22; 2.56) **	2.16 (1.40; 3.35) **
Sexual orientation * Parental autonomy support	-	-	-	-	-	7.24 (1.66; 31.53) **
Sexual orientation * Parental psychological control	-	-	-	-	4.67 (1.07; 20.43) *	-
Traditional victimization * Parental autonomy support	0.08 (1.08, 0.48) *	-	-	-	1.47 (1.05; 2.05) *	-

R^2^ in logistic regression analyses: Nagelkerke R^2^; R^2^ in linear regression analyses: adjusted R^2^. *p* < 0.1; * *p* < 0.05; ** *p* < 0.01; *** *p* < 0.001.

**Table 4:** Regression analyses for negative mental health outcomes and suicidal risk by cyberbullying victimization and sexual orientation. Underline: It was used to more clearly indicate what the difference is between the two tables where one shows traditional bullying and the other cyberbullying.

	Depression	Anxiety	Stress	Suicidal Ideation	Suicidal Plan	Suicidal Attempt
Model information (parsimonious model)	F(6, 945) = 32.02 ***; R^2^ = 0.16	F(5, 955) = 36.31 ***; R^2^ = 0.16	F(8, 945) = 21.10 ***; R^2^ = 0.14	−2 LL = 897.58; χ^2^(3) = 116.10 ***; R^2^ = 0.18	−2 LL = 464.95; χ^2^(5) = 79.94 ***; R^2^ = 0.19	−2 LL = 336.69; χ^2^(4) = 87.06, ***; R^2^ = 0.24
	**β (B, Unstandardized SE)**			**Adjusted Odds Ratio (95% CI)**		
Gender	−0.17 (−2.85, 0.51) ***	−0.17 (−2.40, 0.43) ***	−0.17 (−2.79, 0.49) ***	0.39 (0.28; 0.55) ***	0.49 (0.29; 0.81) **	0.28 (0.14; 0.53) ***
Sexual orientation	0.07 (2.19, 1.00) *	/	0.05 (1.70, 1.01)°	/	/	2.75 (1.18; 6.42) *
Cyberbullying victimization	0.25 (4.72, 0.78) ***	0.25 (4.11, 0.65) **	0.15 (2.69, 0.59) **	2.30 (1.65; 3.22) ***	4.72 (2.78; 8.01) ***	3.32 (2.27; 4.86) ***
**Moderation Effects of Parenting Dimensions**						
Parental autonomy support	−0.08 (−1.05, 0.43) *	−0.10 (−1.02, 0.36) **	/	/	0.80 (0.51; 1.24)	/
Parental psychological control	0.25 (2.88, 0.39) ***	0.24 (2.23, 0.32) ***	0.29 (3.17, 0.34) ***	2.43 (1.95; 3.04) ***	2.18 (1.51; 3.13) ***	2.99 (2.05; 4.36) ***
Cyberbullying victimization * Parental autonomy support	0.09 (1.55, 0.71) *	0.09 (1.40, 0.60) *	/	/	1.85 (1.17; 2.91) **	/
Cyberbullying victimization * sexual orientation	/	/	0.06 (3.16, 2.08)	/	/	/
Sexual orientation * parental psychological control	/	/	0.02 (0.75, 1.37)	/	/	/
Cyberbullying victimization * parental psychological control	/	/	−0.11 (−0.22, 0.64)	/	/	/
Sexual orientation * Cyberbullying victimization * parental psychological control	/	/	0.11 (5.66, 2.08) **	/	/	/

R^2^ in logistic regression analyses: Nagelkerke R^2^; R^2^ in linear regression analyses: adjusted R^2^. ° *p* < 0.1; * *p* < 0.05; ** *p* < 0.01; *** *p* < 0.001.

## Data Availability

Data and syntaxes are available from the authors upon request. Full data are at this point not made publicly available as they form the basis of other manuscripts in preparation.

## Supplementary Materials

The following are available online at https://www.mdpi.com/1660-4601/18/6/2867/s1, Table S1: Full results for the regression analyses for negative mental health outcomes and suicidal risk by traditional and cyberbullying victimization and sexual orientation, Table S2: Full results for the moderation analyses for parenting styles in the relation between traditional bullying victimization and sexual orientation on negative mental health outcomes and suicidal risk, Table S3: Full results for the moderation analyses for parenting styles in the relation between cyberbullying victimization and sexual orientation on negative mental health outcomes and suicidal risk.
