# Supplementary material for: The Moderating Role of Parenting Dimensions in the Association between Traditional or Cyberbullying Victimization and Mental Health among Adolescents of Different Sexual Orientation
Source: Int J Environ Res Public Health. 2021 Mar 11;18(6):2867. doi: 10.3390/ijerph18062867 (PMC8001163; doi:10.3390/ijerph18062867)
Supplement: Supplementary file 1 [file ijerph-18-02867-s001.pdf]

Supplementary material

Table S1. Regression analyses for negative mental health outcomes and suicidal risk by traditional and cyberbullying victimization and sexual orientation

	Depression	Anxiety	Stress	Suicidal ideation	Suicidal plan	Suicidal attempt
**Model information**	F(7, 918)=21.01, p<0.001; R^2=0.13	F(7, 919)=18.57, p<0.001; R^2=0.12	F(7, 920)=13.08, p<0.001; R^2=0.08	-2LL= 905.87; $\chi^2(7)$=79.21, p<.001; R^2=0.13	-2LL= 473.64; $\chi^2(7)$=61.39, p<.001; R^2=0.15	-2LL= 331.46; $\chi^2(7)$=78.44, p<.001; R^2=0.23
	**β (B, UNSTANDARDIZED SE)**			**ADJUSTED ODDS RATIO (95% CI)**		
Age	0.11 (0.49, 0.15)**	0.08 (0.30, 0.12)*	0.10 (0.42, 0.14)**	1.01 (0.92; 1.10)	0.97 (0.85; 1.12)	0.99 (0.83; 1.19)
Gender (ref. girls)	-0.16 (-2.67, 0.53)***	-0.16 (-2.24, 0.44)***	-0.17 (-2.73, 0.52)***	0.43 (0.30; 0.60)***	0.42 (0.25; 0.72)***	0.26 (0.13; 0.52)***
Sexual orientation (ref. heterosexual youth)	0.08 (2.64, 1.06)*	0.08 (2.07, 0.87)*	0.08 (2.38, 1.02)*	1.79 (0.99; 3.26)°	2.66 (1.22; 5.84)*	4.24 (1.76; 10.24)**
Traditional victimization	0.23 (2.67, 0.43)***	0.20 (1.86, 0.35)***	0.15 (1.58, 0.41)***	1.86 (1.48; 2.36)***	1.68 (1.28; 2.20)***	2.03 (1.51; 2.74)***
Traditional victimization * sexual orientation (ref. heterosexual youth)	-0.03 (-0.95, 1.29)	-0.03 (-0.77, 1.07)	-0.00 (-0.05, 1.25)	0.79 (0.39; 1.59)	0.60 (0.27; 1.31)	0.57 (0.26; 1.22)
Cybervictimization	0.17 (3.03, 0.65)***	0.18 (2.57, 0.53)***	0.14 (2.41, 0.62)***	1.52 (1.05; 2.19)*	2.13 (1.43; 3.16)***	2.12 (1.39; 3.24)***
Cybervictimization * sexual orientation (ref. heterosexual youth)	-0.03 (-1.68, 2.29)	-0.01 (-0.35, 1.89)	-0.04 (-2.29, 2.21)	0.60 (0.18; 1.97)	0.88 (0.24; 3.22)	0.82 (0.22; 3.04)
**Parsimonious model results**						
**Model information**	F(5, 920)=28.88, p<0.001; R^2=0.13	F(5, 921)=25.81, p<0.001; R^2=0.12	F(5, 922)=17.98, p<0.001; R^2=0.08	-2LL= 967.02; $\chi^2(3)$=85.81, p<.001; R^2=0.13	-2LL= 504.91; $\chi^2(3)$=60.46, p<.001; R^2=0.14	-2LL= 352.27; $\chi^2(4)$=81.18, p<.001; R^2=0.22
	**β (B, UNSTANDARDIZED SE)**			**ADJUSTED ODDS RATIO (95% CI)**		
Age	0.10 (0.46, 0.14)**	0.08 (0.29, 0.12)*	0.09 (0.39, 0.14)**	/	/	/

Gender	-0.15 (-2.64, 0.53)***	-0.16 (-2.22, 0.44)***	-0.17 (-2.73, 0.51)***	0.43 (0.31; 0.60)***	0.49 (0.30; 0.81)***	0.28 (0.14; 0.55)***
Sexual orientation	0.07 (2.38, 1.04)*	0.07 (1.91, 0.86)*	0.07 (2.26, 1.01)*	/	/	2.65 (1.09; 6.46)*
Traditional victimization	0.22 (2.51, 0.40)***	0.18 (1.75, 0.33)***	0.14 (1.52, 0.39)***	1.90 (1.54; 2.35)***	1.69 (1.33; 2.13)***	1.98 (1.53; 2.56)***
Cybervictimization	0.16 (2.88, 0.62)***	0.17 (2.53, 0.51)***	0.13 (2.21, 0.60)***	1.41 (0.99; 2.00)°	2.01 (1.38; 2.92)***	1.93 (1.29; 1.29)**

Table S2. Moderation analyses for parenting styles in the relation between traditional bullying victimization and sexual orientation on negative mental health outcomes and suicidal risk

	Depression	Anxiety	Stress	Suicidal ideation	Suicidal plan	Suicidal attempt
**Model information (non-parsimonious model)**	F(13, 882)=17.17, p<0.001; R²=0.19	F(13, 883)=14.85, p<0.001; R²=0.17	F(13, 883)=12.96, p<0.001; R²=0.15	-2LL= 802.10; χ²(13)=148.85, p<.001; R²=0.23	-2LL= 426.38; χ²(13)=88.35, p<.001; R²=0.22	-2LL= 294.80; χ²(13)=105.39, p<.001; R²=0.31
	**β (B, UNSTANDARDIZED SE)**			**ADJUSTED ODDS RATIO (95% CI)**		
Age	0.06 (0.28, 0.15)°	0.04 (0.15, 0.12)	0.05 (0.23, 0.14)	0.96 (0.86; 1.06)	0.91 (0.77; 1.07)	0.92 (0.75; 1.12)
Gender	-0.18 (-3.07, 0.52)***	-0.18 (-2.55, 0.44)***	-0.19 (-3.02, 0.51)***	0.35 (0.25; 0.51)***	0.37 (0.22; 0.64)***	0.21 (0.10; 0.44)***
Sexual orientation	0.04 (1.45, 1.09)	0.04 (1.18, 0.91)	0.04 (1.10, 1.06)	1.12 (0.46; 2.72)	0.76 (0.15; 3.95)	3.03 (0.82; 11.20)°
Traditional victimization	0.30 (3.40, 0.45)***	0.23 (2.20,0.37)***	0.18 (1.94, 0.44)***	2.24 (1.70; 2.94)***	2.29 (1.71; 3.05)***	2.68 (1.93; 3.71)***
Traditional victimization * sexual orientation	-0.00 (-0.11, 1.24)	0.05 (1.07, 1.09)	0.05 (1.26, 1.20)	0.99 (0.42; 2.34)	0.86 (0.32; 2.31)	0.66 (0.25; 1.74)
**Moderation effects parenting styles**						
Parental autonomy support	-0.08 (-0.94, 0.46)*	-0.08 (-0.86, 0.38)*	-0.06 (-0.68, 0.45)	0.82 (0.59; 1.13)	0.69 (0.43; 1.12)	0.42 (0.23; 0.77)**
Parental psychological control	0.23 (2.61, 0.42)***	0.22 (2.05, 0.35)***	0.24 (2.63, 0.41)***	2.17 (1.62; 2.91)***	1.97 (1.30; 3.00)**	2.03 (1.21; 3.41)**

Sexual orientation * Parental autonomy support	0.00 (0.01, 1.61)	-0.01 (-0.23, 1.35)	-0.01 (-0.18, 1.57)	2.08 (0.63; 6.84)	3.70 (0.63; 21.90)	15.72 (2.13; 115.78)**
Sexual orientation * Parental psychological control	0.06 (2.30, 1.58)	0.04 (1.43, 1.32)	0.05 (1.79, 1.54)	3.25 (0.88; 12.00)°	9.23 (1.34; 63.66)*	3.79 (0.74; 19.43)
Traditional victimization * Parental autonomy support	0.08 (1.10, 0.54)*	0.00 (0.05, 0.45)	0.02 (0.23, 0.53)	1.25 (0.93; 1.71)	1.43 (0.94; 2.11)°	1.31 (0.88; 1.95)
Traditional victimization* Parental psychological control	-0.03 (-0.33, 0.54)	-0.03 (-0.28, 0.40)	-0.05 (-0.62, 0.47)	0.86 (0.65; 1.13)	0.85 (0.61; 1.17)	0.87 (0.63; 1.20)
Sexual orientation * traditional victimization * parental autonomy support	0.02 (0.84, 1.34)	0.08 (2.37, 1.29)	0.09 (2.91, 1.50)°	1.12 (0.30; 4.18)	1.80 (0.14; 22.66)	2.17 (0.20; 23.50)
Sexual orientation * traditional victimization * parental psychological control	-0.02 (-0.62, 1.07)	-0.04 (-0.90, 0.89)	0.00 (0.09, 1.04)	0.87 (0.40; 1.89)	0.92 (0.30; 2.92)	1.45 (0.43; 4.82)
**Parsimonious model results**						
**Model information**	$F(5, 953)=46.93$, $p<0.001$; $R^2=0.19$	$F(4, 955)=49.49$, $p<0.001$; $R^2=0.17$	$F(3, 958)=55.33$, $p<0.001$; $R^2=0.15$	-2LL= 875.21; $\chi^2(3)=143.50$, $p<.001$; $R^2=0.21$	-2LL= 468.89; $\chi^2(7)=79.56$, $p<.001$; $R^2=0.18$	-2LL= 320.89; $\chi^2(6)=102.71$, $p<.001$; $R^2=0.29$
	**β (B, UNSTANDARDIZED SE)**			**ADJUSTED ODDS RATIO (95% CI)**		
Gender	-0.17 (-2.97, 0.50)***	-0.18 (-2.54, 0.42)***	-0.18 (-2.89, 0.49)***	0.36 (0.25; 0.51)***	0.45 (0.27; 0.75)**	0.24 (0.12; 0.47)***
Sexual orientation	/	/	/	/	0.55 (0.13; 2.32)	3.43 (1.41; 8.38)**
Traditional victimization	0.30 (3.40, 0.39)***	0.23 (2.18, 0.29)***	0.17 (1.87, 0.33)***	2.07 (1.68; 2.55)***	2.20 (1.70; 2.83)**	2.17 (1.70; 2.76)**

**Moderation effects parenting styles**						
Parental autonomy support	-0.08 (-1.00, 0.42)*	-0.09 (-0.94, 0.35)**	/	/	0.74 (0.49; 1.14)	0.52 (0.31; 0.88)*
Parental psychological control	0.24 (2.74, 0.38)***	0.23 (2.18, 0.32)***	0.29 (3.19, 0.33)***	2.32 (1.86; 2.90)***	1.76 (1.22; 2.56)**	2.16 (1.40; 3.35)**
Sexual orientation * Parental autonomy support	/	/	/	/	/	7.24 (1.66; 31.53)**
Sexual orientation * Parental psychological control	/	/	/	/	4.67(1.07; 20.43)*	/
Traditional victimization * Parental autonomy support	0.08 (1.08, 0.48)*	/	/	/	1.47 (1.05; 2.05)*	/

Controlled for main effects of age, gender, sexual orientation and victimization. R^2 in logistic regression analyses: Nagelkerke R^2; R^2 in linear regression analyses: adjusted R^2. ° p<.1; * p<0.05; ** p<.01; *** p<.001

Table S3. Moderation analyses for parenting styles in the relation between cyberbullying victimization and sexual orientation on negative mental health outcomes and suicidal risk

	Depression	Anxiety	Stress	Suicidal ideation	Suicidal plan	Suicidal attempt
**Model information (non-parsimonious model)**	F(7, 918)=21.01, p<0.001; R^2=0.13	F(7, 919)=18.57, p<0.001; R^2=0.12	F(13, 881)=13.17, p<0.001; R^2=0.15	-2LL= 822.06; $\chi^2(13)$=125.39, p<.001; R^2=0.20	-2LL= 420.26; $\chi^2(13)$=94.12, p<.001; R^2=0.23	-2LL= 294.01; $\chi^2(13)$=106.06, p<.001; R^2=0.31
	**β (B, UNSTANDARDIZED SE)**			**ADJUSTED ODDS RATIO (95% CI)**		
Age	0.05 (0.22, 0.15)	0.03 (0.10, 0.12)	0.04 (0.19, 0.14)	0.95 (0.85; 1.05)	0.90 (0.77; 1.06)	0.90 (0.74; 1.11)
Gender	-0.17 (-2.85, 0.53)***	-0.17 (-2.40, 0.44)***	-0.18 (-2.85, 0.51)***	0.40 (0.28; 0.57)***	0.44 (0.25; 0.76)**	0.27 (0.13; 0.55)***
Sexual orientation	0.07 (2.22, 1.10)*	0.06 (1.65, 0.91)°	0.04 (1.41, 1.06)	1.52 (0.71; 3.25)	1.05 (0.26; 4.27)	3.04 (0.89; 10.42)°

Cyberbullying victimization	4.56 (0.81, 0.24)***	0.25 (3.91, 0.67)***	0.20 (3.59, 0.77)***	2.70 (1.67; 4.39)***	4.24 (2.49; 7.24)***	4.26 (2.38; 7.61)***
Cyberbullying victimization * sexual orientation	0.10 (5.48, 4.06)	0.11 (4.85, 3.35)	0.03 (1.54, 3.89)	1.78 (0.19; 16.77)	0.52 (0.01; 19.21)	0.39 (0.01; 13.77)
**Moderation effects parenting styles**						
Parental autonomy support	-0.08 (-1.02, 0.47)*	-0.09 (-0.91, 0.39)*	-0.06 (-0.75, 0.45)°	0.81 (0.59. 1.11)	0.74 (0.45; 1.20)	0.44 (0.24; 0.81)**
Parental psychological control	0.24 (2.80, 0.43)***	0.23 (2.14, 0.35)***	0.25 (2.70, 0.41)***	2.22 (1.67; 2.96)***	1.98 (1.30; 3.02)**	2.03 (1.23; 3.36)**
Sexual orientation * Parental autonomy support	0.01 (0.14, 1.72)	-0.01 (-0.50, 1.42)	-0.02 (-0.73, 1.65)	1.87 (0.59; 6.01)	2.61 50.40. 17.04)	10.50 (1.17; 94.56)*
Sexual orientation * Parental psychological control	0.03 (1.06, 1.64)	-0.00 (-0.14, 1.35)	0.02 (0.63, 1.57)	2.07 (0.61; 7.00)	5.48 (0.91; 33.18)°	2.54 (0.46; 14.02)
Cyberbullying victimization * Parental autonomy support	0.08 (1.34, 0.78)°	0.08 (1.12, 0.64)°	0.08 (1.36, 0.75)°	1.38 (0.86; 2.22)	2.01 (1.10; 3.70)*	1.70 (0.94. 3.07)°
Cyberbullying victimization* Parental psychological control	0.03 (0.66, 0.68)	0.01 (0.19, 0.56)	0.01 (0.16, 0.65)	1.12 (0.72; 1.73)	1.33 (0.72; 2.50)	1.23 (0.75; 2.04)
Sexual orientation * Cyberbullying victimization * parental autonomy support	0.09 (3.75, 4.09)	0.05 (1.77, 3.38)	-0.05 (-1.87, 3.93)	1.24 (0.25; 6.03)	0.15 (0.00; 24.52)	0.08 (0.00; 14.41)
Sexual orientation * Cyberbullying victimization * parental psychological control	0.00 (0.05, 3.31)	0.07 (3.09, 2.73)	0.12 (6.16, 3.17)°	0.76 (0.12; 4.79)	4.14 (0.03; 513.20)	10.79 (0.07; 1572.66)

**Parsimonious model results**						
**Model information**	F(6, 945)=32.02, p<0.001; R²=0.16	F(5, 955)=36.31, p<0.001; R²=0.16	F(8, 945)=21.10, p<0.001; R²=0.14	-2LL= 897.58; χ²(3)=116.10, p<.001; R²=0.18	-2LL=464.95; χ²(5)= 79.94, p<.001; R²=0.19	-2LL= 336.69; χ²(4)=87.06, p<.001; R²=0.24
	**β (B, UNSTANDARDIZED SE)**			**ADJUSTED ODDS RATIO (95% CI)**		
Gender	-0.17 (-2.85, 0.51)***	-0.17 (-2.40, 0.43)***	-0.17 (-2.79, 0.49)***	0.39 (0.28; 0.55)***	0.49 (0.29; 0.81)**	0.28 (0.14; 0.53)***
Sexual orientation	0.07 (2.19, 1.00)*	/	0.05 (1.70, 1.01)°	/	/	2.75 (1.18; 6.42)*
Cyberbullying victimization	0.25 (4.72, 0.78)***	0.25 (4.11, 0.65)**	0.15 (2.69, 0.59)**	2.30 (1.65; 3.22)***	4.72 (2.78; 8.01)***	3.32 (2.27; 4.86)***
**Moderation effects parenting styles**						
Parental autonomy support	-0.08 (-1.05, 0.43)*	-0.10 (-1.02, 0.36)**	/	/	0.80 (0.51; 1.24)	/
Parental psychological control	0.25 (2.88, 0.39)***	0.24 (2.23, 0.32)***	0.29 (3.17, 0.34)***	2.43 (1.95; 3.04)***	2.18 (1.51; 3.13)***	2.99 (2.05; 4.36)***
Cyberbullying victimization * Parental autonomy support	0.09 (1.55, 0.71)*	0.09 (1.40, 0.60)*	/	/	1.85 (1.17; 2.91)**	/
Cyberbullying victimization * sexual orientation	/	/	0.06 (3.16, 2.08)	/	/	/
Sexual orientation * parental psychological control	/	/	0.02 (0.75, 1.37)	/	/	/
Cyberbullying victimization * parental psychological control	/	/	-0.11 (-0.22, 0.64)	/	/	/
Sexual orientation * Cyberbullying	/	/	0.11 (5.66, 2.08)**	/	/	/

victimization * parental psychological control	

Controlled for main effects of age, gender, sexual orientation and victimization. R^2 in logistic regression analyses: Nagelkerke R^2; R^2 in linear regression analyses: adjusted R^2. ° $p<.1$; * $p<0.05$; ** $p<.01$; *** $p<.001$
